# Mask-Induced Facial Dermatoses in the Saudi Arabian Population During the COVID-19 Pandemic: A Cross-Sectional Study

**DOI:** 10.7759/cureus.31151

**Published:** 2022-11-06

**Authors:** Halah O Alamawi, Maram S Alruwaili, Sarah K Alswayed, Wareef A Alhumaidi, Safiah O Aldabali, Haifa A Alfalah

**Affiliations:** 1 College of Medicine, Princess Nourah Bint Abdul Rahman University, Riyadh, SAU; 2 College of Medicine, Jouf University, Sakaka, SAU; 3 College of Medicine, King Saud Bin Abdulaziz University for Health Sciences, Riyadh, SAU; 4 College of Medicine, Taif University, Taif, SAU; 5 Al-Qunfudhah College of Medicine, Umm Al-Qura University, Al-Qunfudhah, SAU; 6 Dermatology Division, Internal Medicine and Critical Care Department, King Abdullah Bin Abdulaziz University Hospital, Riyadh, SAU

**Keywords:** health-care workers, mask, mask-induced dermatosis, sars-cov-2, covid-19

## Abstract

Background

The severe acute respiratory syndrome coronavirus 2 (SARS-COV-2) emerged in 2019 and was responsible for noteworthy morbidity and death throughout the world. Due to preventive measures, various adverse reactions to the skin occurred which were associated with prolonged use of wearing a face mask.

Objectives

The study aimed to determine the incidence and assess the clinical features of mask-induced dermatoses.

Methods

A cross-sectional study was conducted involving both healthcare and non-healthcare individuals in Saudi Arabia. A questionnaire was designed that included mask-related problems, preexisting skin conditions, frequency and duration of use of face masks, type of face mask, and demographic information. Further information on their clinical symptoms was collected.

Results

This study included 2326 participants. Participants who refused to participate in the study and did not wear masks (232) were excluded from the study. Redness, itchiness, and acne were the most reported symptoms. 37.8% of the total wore the mask daily with 58.2% using their face mask for more than two hours per day. 44.4% of the participants had mask-induced dermatosis. Almost half of the participants (46.8%) had the cheek as the most affected area. Contact dermatitis was significantly less in non-healthcare workers as compared to healthcare workers (p<0.001). Similarly, conditions like nonspecific erythema (p=0.004) and rosacea (p=0.027) were also significantly less in non-healthcare workers as compared to healthcare workers.

Conclusion

There was a strong relationship between the frequency of mask use and facial dermatosis during the pandemic. The prevalence or pattern of mask-induced facial dermatoses was not significantly different between healthcare workers and non-healthcare workers. However, contact dermatitis and nonspecific erythema were significantly more common in healthcare workers.

## Introduction

Coronaviruses were first discovered in humans in 1962 causing upper respiratory tract infections and later it was discovered that they can affect the lower respiratory tract. Coronaviruses fit in the coronavirus family which has multiple subtypes known as alpha, beta-gamma, and delta types viruses. The severe acute respiratory syndrome coronavirus 2 (SARS-CoV-2) is a beta type of coronavirus [1].

At the end of 2019, an infectious respiratory pandemic began in the district city of Wuhan, China, and was known as SARS-CoV-2 or coronavirus disease 2019 (COVID-19). Globally, 3.5 million people have been infected with this virus which has caused the mortality of more than 0.24 million people around the globe [2].

The virus itself has emerged from cross-species transmission to humans like its predecessors. Once the outbreak occurred, it primarily transported itself from one human to another through the small respiratory droplets of the infected person. The droplets can be exhaled by a cough/sneeze of the infected person and can be transmitted via direct contact with the mucosal membrane of the healthy person. However, the droplets are not able to pass more than 6 feet. Various studies further suggest that it can also be transmitted via fecal or blood swabs [3]. The incubation time for the disease is 14 days with characteristic symptoms like fever, sore throat, dry cough, GI upset, and loss of sense of smell and taste [4].

After the incidence of the pandemic, one of the key approaches taken to prevent the transmission of this disease was to use personal protective equipment (PPE) i.e., face masks, and face helmets as the shield for the protection of respiratory symptoms among healthcare workers (HCWs) as well as the local population. This period of use of PPEs against COVID-19 was prolonged and continued till the end of this pandemic [5]. The extended use of face masks in turn caused multiple skin-related problems like an increase in the progression of existing dermatosis, contact, seborrheic dermatitis, and acne among various health workers [6,7]. Various other studies have provided evidence of facemask-associated damage to the skin as it can trigger rosacea flares and acne as well. Furthermore, a new phenomenon has emerged called mask-induced Koebner [8].

Even though it was mandatory for healthcare professionals throughout the world to use face masks, the authorities have also encouraged individuals of the local population to use them to reduce the transmission of the virus and prevent local outbreaks. Since this use of face masks was applied worldwide, the period of use of face masks also increased which brought attention to the occurrence of face mask-induced dermatosis in the local population [9]. Currently, there are very few research studies present based on a large-scale population on the adverse reaction on the skin due to prolonged use of face masks among the general population. Therefore, the focus of this study was to increase the understanding of the prevalence, clinical characteristics, and treatment options for mask-induced dermatoses among HCWs and non-healthcare workers (non-HCWs).

## Materials and methods

Study design and setting

This is a cross-sectional electronic questionnaire-based study that was distributed via social media.

Study participants

The sample size was determined via Raosoft online calculator. The recommended sample size was 385 with a confidence interval of 95% and a 5% margin of error. However, 2326 people responded to the questionnaire. The study’s inclusion criteria were people over 18 years old, both healthcare workers and non-healthcare workers who live in Saudi Arabia, those who confirmed their agreement, and those who completed the questionnaire. Participants who refused to participate in the study and did not wear masks (232), people not living in Saudi Arabia, those under 18 years old, those who did not confirm their agreement, and those who did not complete the questionnaire were excluded from the study.

Data collection process

To guarantee that each participant was represented correctly, similar links were distributed to each participant. The questionnaire's content was identical across all links. In addition, besides the link provided via social media, a brief introduction of the study's goals and objectives was displayed. The participants were enlightened that participation was completely voluntary and anonymous and that all information would be kept completely private. Following that, participants must confirm their involvement in the study by selecting either "agree" to continue with the study or "disagree". The data collection period was from April to June 2022.

Instrument development

The questionnaire included four parts. The first part was about the participant's demographics, including the participant's place of residence, gender, age, occupation, marital status, and education level. In the second part, participants were asked about how often they wear a facial mask per week, the estimated duration of mask-wearing per day, the type of facial mask, whether the facial mask was fitting perfectly, and if they re-used a disposable mask or not. In the third part, we evaluated the prevalence and presentation of face mask-induced facial dermatoses among individuals. Participants were asked about the skin conditions that they have been diagnosed with, whether they have been diagnosed with these conditions before or after using face masks, was the condition aggravated/worsened by using face masks, how many times did they get mask induced dermatoses during the COVID-19 pandemic, and the facial area that was involved. In the fourth part, we evaluated risk factors associated with the incidence of mask-induced dermatoses. Participants were asked if they had an allergy to a specific type of face mask. In addition, they were asked about their adherence to the general measures that prevent mask use-related facial dermatoses and their skin type. Also, some mask pictures were added to the questionnaire.

This questionnaire was validated using a method that included focus group discussions, expert appraisal, pilot research, reliability, and validity testing. Three dermatology experts and one biostatistician worked together to validate our questionnaire. For reliability and validity analysis, data from a pilot study with 21 individuals were used. The content and face validity of the questionnaire were assessed using expert review and focused group discussion. An exploratory factor analysis was used to examine the questionnaire's construct validity. Internal consistency was examined for questionnaire reliability, and Cronbach's result was 0.81. If the Cronbach's value is more than 0.7, the questionnaire is considered internally consistent. All of the questionnaire items were translated into Arabic by a healthcare practitioner and a translator specialist who is skilled in both Arabic and English. Two more specialists who were fluent in both languages then translated the Arabic questionnaire into English. The back-translated version of the questionnaire was compared to the original English version to confirm the quality of the translation.

Data analysis

The results of the questionnaires were displayed in an Excel version 16.16.23 (Microsoft, Redmond, WA, USA) and data were analyzed statistically using SPSS)version 26 (IBM Corp., Armonk, NY, USA). To test the relationship between variables, qualitative data were expressed as numbers and percentages, and the Chi-squared test (χ2) was used. A p-value of less than 0.05 was considered statistically significant.

Ethical considerations

Ethical approval was provided by the institutional review board (IRB) at Princess Nourah Bint Abdul Rahman University, Riyadh, Saudi Arabia (HAP-01-R-059).

## Results

Table 1 shows that 63.6% of the participants had an age that ranged from 18-25 years, 73.5% were females and 29.7% were married. Also, 81.6% of them had a university level of education or higher and only 9.2% were HCWs. About 26% of the participants were from the Eastern region of Saudi Arabia.

**Table 1:** Distribution of the participants according to their demographic characters (No.:2090)

Variable	No. (%)
Age	
18-25	1330 (63.6)
26-35	385 (18.4)
36-45	236 (11.3)
46-60	133 (6.4)
More than 60	7 (0.3)
Gender	
Female	1537 (73.5)
Male	554 (26.5)
Marital status	
Married	620 (29.7)
Not married	1471 (70.3)
Please select your education level?	
High school or lower	384 (18.4)
University or higher	1707 (81.6)
Please select your occupation?	
Student	1210 (57.9)
Unemployed	215 (10.3)
Retired	40 (1.9)
Employed (healthcare sector)	193 (9.2)
Employed (non-healthcare sector)	433 (20.7)
Health care worker?	
Yes	193 (9.2)
No	1897 (90.8)
Which part of Saudi Arabia do you reside in?	
Northern region	246 (11.8)
Eastern region	552 (26.4)
Southern region	357 (17.1)
Western region	545 (26.1)
Middle region	391 (18.7)

Table 2 shows that 37.8% of the participants were wearing the face mask every day, 29.8% were wearing it for five to seven hours and the majority (86.8%) were using a procedure/surgical mask. About half of the participants (50.4%) reported that the mask was well fit. 29.2% of them reported that they never re-use a disposable mask, and 23.8% mentioned that they do this sometimes.

**Table 2:** Distribution of the participants according to the pattern of using the face mask (No.:2090)

Variable	No. (%)
How often do you wear a face mask per week?	
1-2 days	405 (19.4)
3-4 days	364 (17.4)
5-6 days	531 (25.4)
Every day	791 (37.8)
What is the estimated duration of mask wearing per day?	
Less than two hours	374 (17.9)
2-4 hours	594 (28.4)
5-7 hours	624 (29.8)
8-10 hours	386 (18.5)
12 hours	113 (5.4)
What type of face mask you wear usually?	
Face shield	8 (0.4)
Procedure/surgical mask	1816 (86.8)
Non-woven (e.g.air queen)	60 (2.9)
Cloth mask	160 (.7)
N95	47 (2.2)
Does your face mask usually fit your face perfectly?	
Tight	110 (5.3)
Wide	927 (44.3)
Well fit	1054 (50.4)
Do you usually re-use a disposable mask?	
Never	608 (29.1)
Often	338 (16.2)
Sometimes	498 (23.8)
Always	229 (11)
Rarely	418 (20)

**Figure 1:**
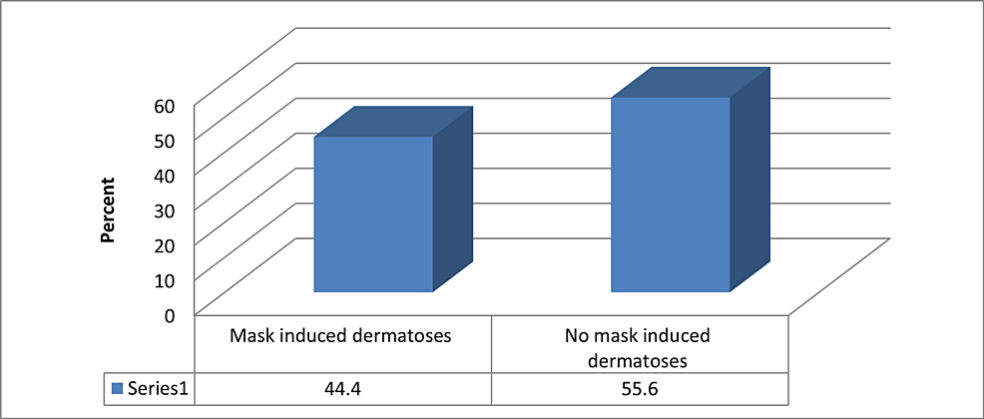
Percentage distribution of mask-induced facial dermatoses among studied participants

Mask-induced facial dermatoses among studied participants were 44.4% (No.:928) (Figure 1).

**Figure 2:**
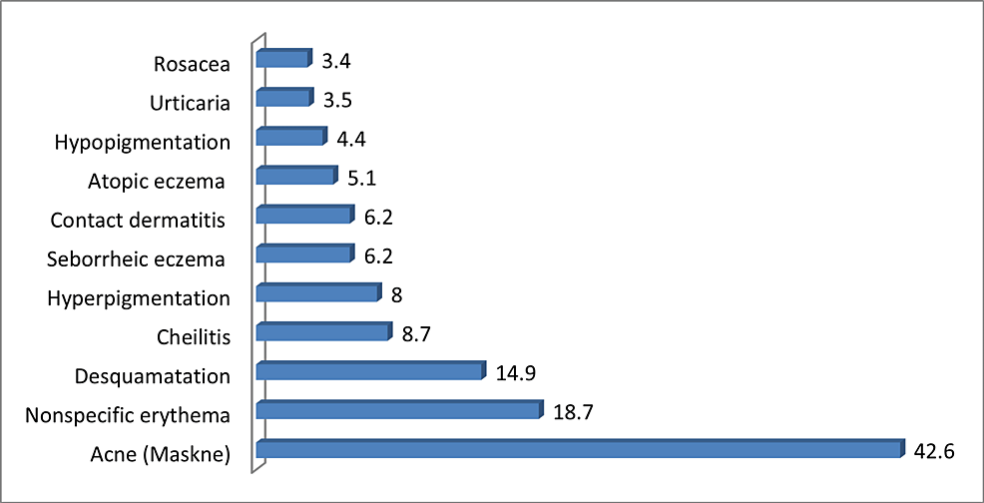
Percentage presentation of face mask-induced facial dermatoses among studied participants

The most common presentation of face mask-induced facial dermatoses was acne (maskne) (42.6%), nonspecific erythema (18.7), and desquamation (14.9%) (Figure 2).

Table 3 shows that among participants having mask-induced dermatoses (No.:928), 80.5% got mask-induced dermatoses during the COVID-19 pandemic one to three times. Most of them (46.8%) had the dermatoses on their cheeks and the most common symptoms were redness (51%), itching (49.5%), acne (43.7%), and blisters (38.6%). The most commonly used measures to prevent mask use-related facial dermatoses were maintaining oral hygiene (66.1%), cleansing skin with a gentle soap-free cleanser (58.6%), and taking regular breaks from the mask to relieve the pressure and prevent moisture build-up (54.2%). Only 44.7% of the participants were allergic to face masks, and the procedure/surgical mask was the most common allergic type (45.4%). Most participants with facial mask-induced dermatosis (51.3%) were cleaning their faces after using facial masks and 31.1% reported applying cosmetics. The most common skin type among them was the combination type.

**Table 3:** Distribution of pattern of mask-induced dermatoses and associated factors (No.:928)

Variable	No. (%)
How many times did you get mask induced dermatoses during COVID pandemic?	
1-3 times	746 (80.5)
3-6 times	105 (11.3)
6 times or more	77 (8.2)
Where do you usually get the dermatoses (in which area in the face)? Please choose what is applicable to your condition,	
Ears	179 (19.2)
Nose	104 (11.2)
Cheeks	435 (46.8)
Chin	210 (22.8)
While having a mask induced dermatosis ,which of the following symptoms did you suffer from?(check what is applicable)	
Itchiness	460 (49.5)
Redness	474 (51)
Blisters	359 (38.6)
Dry/cracked skin	259 (27.9)
Burning sensation	204 (21.9)
Tightness/stinging	194(20.9)
Acne	406 (43.7)
Did you use the following general measures to prevent mask-use related facial dermatoses (You can choose more than one option)	
Cleanse skin with a gentle soap-free cleanser	544 (58.6)
Apply a light emollient at least 30 minutes before applying facial mask	448 (48.2)
Apply a silicon based barrier tape—eg, siltape (Advancis)—to nasal bridge and cheeks	94 (10.1)
Take time to fit the mask and ensure it is not over tight	421 (45.3)
Take regular breaks from the mask (every one hour for respirators) to relieve the pressure and prevent moisture build up	503 (54.2)
Maintain oral hygiene (teeth brushing twice daily and daily interdental flossing/brushing)	614 (66.1)
Are you allergic to specific type of face mask?	
No	514 (55.3)
Yes	414 (44.7)
If yes, what mask you are allergic to? (No.:410)	
Face shield	8 (1.9)
Procedure/surgical mask	188 (45.4)
Non-woven (e.g.air queen)	32 (7.7)
Cloth mask	145 (35)
N95	41 (10)
Do you clean your face after using facial masks?	
No	452 (48.7)
Yes	476 (51.3)
Do you apply any cosmetic products such as (foundations, concealers, face powders, etc) underneath the face mask?	
No	640 (68.9)
Yes	288 (31.1)
What’s your skin type?	
Dry	129 (13.9)
Sensitive	25 (2.6)
Oily	302 (32.5)
Normal	137 (14.7)
Combination (both oily and dry skin)	335 (36.3)

Table 4 demonstrates that HCWs had a significantly higher percentage of those who were wearing the face mask on daily basis, who were wearing it for a longer duration (eight to 10 hours), and who were wearing the N95 mask type (p=< 0.05). 

**Table 4:** Difference between healthcare workers and non-healthcare workers according to pattern of mask use (No.:2090)

Variable	Group		χ2	p-value
	Health care workers No. (%)	Non-heath care workers No. (%)		
How often you wear a face mask per week?				
1-2 days	15 (7.8)	390 (20.6)	33.55	<0.001
3-4 days	20 (10.4)	343 (18.1)		
5-6 days	48 (30.1)	473 (24.9)		
Every day	100 (51.8)	691 (36.4)		
What is the estimated duration of mask wearing per day?				
Less than two hours	13 (6.7)	360 (19)	89.49	< 0.001
2-4 hours	38 (6.4)	556 (29.3)		
5-7 hours	44 (22.8)	580 (30.6)		
8-10 hours	73 (37.8)	313 (16.5)		
12 hours	25 (13)	88 (4.6)		
What type of face mask you wear usually?				
Face shield	1 (0.5)	7 (0.4)	36.12	< 0.001
Procedure/surgical mask	155 (80.3)	1660 (87.5)		
Non-woven (e.g.air queen)	7 (3.6)	53 (2.8)		
Cloth mask	14 (7.3)	146 (7.7)		
N95	16 (8.3)	31 (1.6)		
Does your face mask usually fit your face perfectly?				
Tight	15 (7.8)	95 (5)	4.36	0.113
Wide	75 (38.9)	852 (44.9)		
Well fit	103 (53.4)	950 (50.1)		
Do you usually re-use a disposable mask?				
Never	55 (28.5)	552 (29.1)	0.23	0.993
Often	33 (17.1)	305 (16.1)		
Sometimes	47 (24.4)	451 (23.8)		
Always	20 (10.4)	209 (11)		
Rarely	38 (19.7)	380 (20)		

Table 5 shows that HCWs had a significantly higher percentage of those who were presented with contact dermatitis and nonspecific erythema compared to non-HCWs (p=<0.05). At the same time, HCWs had a significantly higher percentage of those who had a combination skin type (p=<0.05). On the other hand, a non-significant difference was found between HCWs and non-HCWs according to other presentations or the prevalence of mask-induced facial dermatoses (p=>0.05).

**Table 5:** Difference between healthcare workers and non-healthcare workers according to presentation and prevalence of mask-induced facial dermatoses (No.:2090)

Variable	Group		χ2	p-value
	Health care workers No. (%)	Non-heath care workers No. (%)		
Which of the following skin conditions have you been diagnosed with?				
Acne (Maskne)	90 (46.6)	801 (42.2)	1.39	0.238
Hypopigmentation	11 (5.7)	81 (4.3)	0.85	0.356
Hyperpigmentation	19 (9.8)	148 (7.8)	0.99	0.319
Contact dermatitis	24 (12.4)	105 (5.5)	14.4	<0.001
Nonspecific erythema	51 (26.4)	229 (17.9)	8.44	0.004
Desquamatation	30 (15.5)	281 (14.8)	0.07	0.786
Urticaria	9 (4.7)	65 (3.4)	0.78	0.376
Cheilitis	24 (12.4)	158 (8.3)	3.71	0.054
Atopic eczema	15 (7.8)	92 (4.8)	3.08	0.079
Seborrheic eczema	18 (9.3)	112 (5.9)	3.51	0.061
Rosacea	12 (6.2)	60 (3.2)	4.91	0.027
Mask induced dermatosis				
Yes	82 (42.5)	846 (44.6)	0.31	0.574
No	111 (57.5)	1051 (55.4)		
What’s your skin type?				
Dry	11 (5.7)	114 (6)	11.09	0.049
Sensitive	2 (1)	23 (1.2)		
Oily	14 (7.3)	288 (15.2)		
Normal	16 (8.3)	121 (6.4)		
Combination (both oily and dry skin)	39 (20.2)	296 (15.6)		

Table 6 shows that a non-significant difference was found between HCWs and non-HCWs regarding the pattern of mask-induced dermatoses or associated factors (p=>0.05).

**Table 6:** Difference between healthcare workers and non-healthcare workers according to pattern of mask-induced dermatoses and associated factors (No.:928)

Variable	Group		χ2	p-value
	Health care workers No. (%)	Non-heath care workers No. (%)		
How many times did you get mask induced dermatoses during COVID pandemic?				
1-3 times	68 (35.2)	678 (35.7)	0.98	0.806
3-6 times	7 (3.6)	98 (5.2)		
6 times or more	7 (3.6)	70 (3.7)		
Where do you usually get the dermatoses (in which area in the face)? Please choose what is applicable to your condition				
Ears	16 (8.3)	163 (8.6)	0.49	0.974
Nose	9 (4.7)	95 (5)		
Cheeks	37 (19.2)	398 (21)		
Chin	20 (10.4)	190 (10)		
While having a mask induced dermatosis, which of the following symptoms did you suffer from?(check what is applicable)				
Itchiness	45 (23.3)	15 (21.9)	1.29	0.525
Redness	42 (21.8)	432 (22.8)	0.31	0.854
Blisters	36 (18.7)	323 (17)	0.13	0.52
Dry/cracked skin	29 (15)	230 (12.1)	0.27	0.259
Burning sensation	20 (10.4)	184 (9.7)	0.6	0.738
Tightness/stinging	21 (10.9)	173 (9.1)	1.47	0.479
Acne	33 (17.1)	373 (19.7)	0.74	0.688
Are you allergic to specific type of face mask?				
No	48 (24.9)	466 (24.6)	0.66	0.718
Yes	34 (17.6)	380 (20)		
If yes, what mask you are allergic to? (No.:410)				
Face shield	1 (0.5)	7 (0.4)	3.29	0.655
Procedure/surgical mask	15 (7.8)	173 (9.1)		
Non-woven (e.g.air queen)	2 (1)	30 (1.6)		
Cloth mask	10 (5.2)	135 (7.1)		
N95	6 (3.1)	35 (1.8)		
Did you use the following general measures to prevent Mask-use related facial dermatoses (You can choose more than one option)				
Cleanse skin with a gentle soap-free cleanser	49 (25.4)	459 (26.1)	0.36	0.835
Apply a light emollient at least 30 minutes before applying facial mask	40 (20.7)	408 (21.5)	0.32	0.85
Apply a silicon-based barrier tape—e.g., siltape (Advancis)—to nasal bridge and cheeks	11 (5.7)	83 (4.4)	1.34	0.512
Take time to fit the mask and ensure it is not over tight	37 (19.2)	384 (20.2)	0.31	0.853
Take regular breaks from the mask (every one hour for respirators) to relieve the pressure and prevent moisture build up	48 (24.9)	455 (24)	0.97	0.616
Maintain oral hygiene (teeth brushing twice daily and daily interdental flossing/brushing)	53 (27.5)	561 (29.6)	0.4	0.816
Do you clean your face after using facial masks?				
No	41 (21.2)	407 (21.5)	0.33	0.845
Yes	41 (21.2)	435 (22.9)		
Do you apply any cosmetic products such as (foundations, concealers, face powders, etc) underneath the face mask?				
No	56 (29)	580 (30.6)	0.26	0.875
Yes	26 (13.5)	262 (13.8)		

## Discussion

One of the most effective protections against COVID-19 is wearing a mask. Therefore, wearing a face mask in public areas is required in many countries including Saudi Arabia [10]. Previous studies have revealed that wearing a face mask for a prolonged period of time promotes friction, occlusion, and hyperhidrosis, and this compromises the skin and epidermal integrity and appears as facial dermatosis [11]. The most frequent adverse effects of face masks include itching, stinging, and dryness. Additionally, contact dermatitis and acne are the two most common skin diseases [12]. In this study, we aimed to assess face mask-induced facial dermatoses in terms of clinical presentation and factors associated with mask use among the Saudi population during the COVID-19 pandemic.

One of the research aspects was to assess mask-wearing habits among participants. Among 2326 participants, 37.8% wore their masks daily, and 25.4% used their masks five to six days a week. Moreover, most participants (58.2%) used their face masks for more than two hours per day. 29.8% of them used masks between five and seven hours per day. These findings suggest that most of the participants used masks for most of the week for long hours and this would increase the risk of mask-induced dermatosis and its complications [13]. Furthermore, concerning results demonstrates that 51% of the participants re-used their masks regularly and 23.8% reported that they "sometimes" re-used a disposable mask, only 29.1% of the respondents answered that they "never" re-used a disposable face mask. Centers for Disease Control and Prevention (CDC) guidelines do not recommend reusing disposable face masks [14]. Re-used disposable face masks can contain dead skin cells, debris, microbe, and sweat or might be improperly stored which can be a source of contamination and infection [15,16]. When comparing mask use patterns between HCWs and non-HCWs, results showed that over half of HCWs (51.8%) used the face mask daily and they are less likely to wear the face mask for a lesser frequency per week. Only 7.8% of them used their masks one to two times per week when comparing them to non-HCWs (20.6%). This is probably due to the nature of their work, and a higher level of knowledge of the face mask's role in infection prevention [17]. Moreover, the face mask-wearing duration was longer in HCWs in comparison to non-HCWs. Among HCWs, 50.8% wore a mask for over eight hours per day. This could be due to longer working hours among HCWs.

In this study, 44.4% of the study participants had mask-induced dermatosis and that is similar to a previous study done by Bukhari et al. in Saudi Arabia in which they found that 48.6% had dermatological manifestations associated with face mask use [10]. Furthermore, when observing the overall prevalence of face mask-induced dermatosis, it was noted that 42.6% of participants had masks followed by nonspecific erythema (18.7%). Maskne probably was the commonest presentation since acne on its own is one of the most common dermatological diseases worldwide [11]. Nonspecific erythema might be associated with mask friction on the face and inflammatory processes [18]. Maskne was also the highest presentation associated with mask-induced dermatosis in previous research such as the one done by Althobaiti et al. [19] but differs from a study done by Choi et al. in South Korea in which their most common presentation was found to be contact dermatitis [12]. In our study, maskne was the commonest presentation among both HCWs and non-HCWs (46.6% and 42.2%, respectively). Contact dermatitis (12.4%) and nonspecific erythema (26.4%) were more prevalent among HCWs than non-HCWs.

Most affected face parts and dermatosis distribution were assessed among participants. Results showed that almost half of the participants (46.8%) reported that the cheeks were the most affected face part, and the least is the nose (11.2%). This finding is similar to many previous studies like the ones done by Althobaiti et al. and Bakhsh et al. [19,20]. Furthermore, symptoms associated with mask-induced dermatosis were evaluated in this research. The three most reported symptoms were redness (51%), itchiness (49.5%), and acne (43.7%). The least reported symptoms were tightness or stinging. These outcomes are congruent with the most reported presentations among the studied participants which were reported to be acne and nonspecific erythema. These findings are slightly different from previous research done by Bukhari et al. and Althobaiti et al. [10,19]. Almost half of the participants did follow the preventative measure to prevent mask-induced dermatosis with oral hygiene (66.1%) being the most applied preventative measure and silicon-based barrier tape being the least followed preventative measure. This might be due to the lesser popularity of silicon-based barrier tape among the general population. Moreover, 45.4% reported that surgical/procedural masks induced allergies. This could be because surgical/procedural masks were the most commonly used masks among our population (86.8%).

Among the studied participants, 36.3% had a combination skin type and 32.5% had oily skin. It is suggested that participants' skin type had a role in developing acne as acne was the most common dermatosis among the current study participants [21]. This is partially similar to the research done by Althobaiti et al. as the commonest skin types among their participants were combination and normal skin [19]. The possible limitation of this study is that the majority of the participants were females and only 9.2% of the participants were HCWs.

## Conclusions

In conclusion, our paper studied mask-induced dermatosis from different aspects including associated demographic features, face mask-use pattern, prevalence, presentation, disease characteristics, and associated factors. There was a strong link between the frequency of mask use and facial dermatosis with redness, itching, and acne being the most prevalent symptoms. Moreover, across all mask varieties, the surgical mask was the main cause of allergy. Additionally, contact dermatitis and non-specific erythema were more common in HCWs. Therefore, future studies on prevention/treatment, the establishment of diagnostic criteria related to mask-induced dermatosis, and exploring the dermatosis concerning the type of mask materials are needed.
